# Exploring the twin potential of nanostructured TiO_2_:SeO_2_ as a promising visible light photocatalyst and selective fluorosensing platform

**DOI:** 10.1038/s41598-024-64167-5

**Published:** 2024-06-13

**Authors:** Aarti Sharma, Nidhi Sharotri, Pankaj Kandwal, Rakesh Kumar Sharma, Dhiraj Sud, Ritu Rai, Anna Hnydiuk-Stefan

**Affiliations:** 1 Department of Chemistry, Sant Longowal Institute of Engineering and Technology (Deemed University), Longowal, 148106 Sangrur, Punjab India; 2 Department of Chemistry, NIT Uttarakhand, Srinagar, Garhwal, 246174 India; 3 Department of Chemistry, University of Delhi, Delhi, 110007 India; 4 Department of Process and Environmental Engineering, Opole University of Technology, Ul. Prószkowska 76, 45-758 Opole, Poland; 5 Faculty of Production Engineering and Logistics, Opole University of Technology, Ul. Prószkowska 76, 45-758 Opole, Poland

**Keywords:** TiO_2_:SeO_2_ nanostructure, Photocatalyst, Fluorophore, Static quenching, Aromatic pollutant, Catalysis, Environmental chemistry, Chemical synthesis, Theoretical chemistry

## Abstract

The present work describes the development of TiO_2_/SeO_2_ nanostructure as a potential candidate for visible light photocatalysis as well as selective fluorophore for the sensing of picric acid. The obtained nanostructure consists of uniform globular nanoparticles having approximate size of 170 nm and possess an optical band gap of 2.33 eV with absorption maxima at 473 nm. The photocatalyst was able to achieve 90.34% degradation efficiency for 2, 4-dichlorophenol (2,4-DCP) with rate constant of 0.0046 min^−1^ in the visible region. Further the nanostructure was able to serve as a selective fluorophore for sensing of Picric acid portraying more than 95% of fluorescence quenching when the concentration of PA is 10^–4^ M. Theoretical calculations indicate the interaction of organic pollutants with the nanostructure and reveal that both picric acid (− 66.21 kcal/mol) and 2,4-DCP (− 12.31 kcal/mol) possess more negative binding energy values demonstrating a strong interaction of both with the nanostructure, making it suitable for the degradation as well as sensing of organic pollutants. Thus this study explains the potential of prepared catalyst for waste water treatment.

## Introduction

Environmental pollution is currently a significant global issue, resulting in widespread harm to life on our planet^1,2^. Water pollution arises from the emission of toxic organic substances, including dyes, acids, and antibiotics, among others, from textile, chemical, and pharmaceutical industries into drinkable water sources, such as rivers, lakes, and ponds. A significant proportion of organic substances possess carcinogenic properties. Furthermore, the contamination of water inherently results in the contamination of soil, hence exerting a direct or indirect impact on various aspects of daily existence^3,4^. Taking into account the potential long-term impact of effluent toxicity on the environment, it is crucial to employ efficient and effective water treatment techniques to eliminate or treat industrial waste^5^. Various water treatment techniques have been utilized thus far, including biodegradation, coagulation, adsorption, and photocatalysis, among others, to eliminate organic pollutants^6^. One of the approaches that has demonstrated reliability in the removal of toxic pollutants or the conversion of toxic pollutants into less hazardous pollutants is the photocatalysis method^7^. This approach has numerous benefits, such as its eco-friendliness, absence of secondary pollutants, cost-efficiency, and reusability of the catalyst^8^. Despite the considerable progress achieved in the field of photocatalysis, the practical utilization of pure photocatalysts under solar light is hindered by two primary constraints. Initially, a number of unadulterated photocatalysts possessing broad energy ranges can alone undergo activation through ultraviolet light which constitutes less than 5% of the total solar spectrum^9^. Furthermore, it is highly probable that the photo generated electron–hole pairs of individual semiconductor photocatalysts would undergo recombination, resulting in a decrease in both quantum and photocatalytic efficiencies^10^. In recent decades, researchers have devised several techniques to address the limitations of pristine photocatalysts. These efforts include element doping^11^, semiconductor coupling^12,13^, dye sensitization^14^ and heterostructured nanostructured composites^15–18^. Semiconductor coupling and nanocomposites formation have emerged as prominent areas of research in the field of photocatalysis due to its ability to facilitate the integration of heterojuction systems, hence enhancing the efficiency of e^−^ and h^+^ separation^19–21^. Though lot of candidates have been exposed by implementing modifications in pristine photocatalysts for carrying out the photocatalytic degradation of range of organic pollutants yet TiO_2_ has been the most popular semiconductor photocatalyst as compared to others due to its prenominal characteristics such as high photocatalytic activity, chemical and biological stability, insolubility in water, acid and base environments, resistance to corrosion, lack of toxicity, affordability, and availability. But there are a few downsides to TiO_2_'s practical application that outweigh all the benefits. Most importantly the photoactive behavior of TiO_2_ is only witnessed when exposed to ultraviolet light followed. Transforming TiO_2_'s optical response from the ultraviolet to the visible light area can greatly enhance the material's photocatalytic activity. The visible light response of TiO_2_'s photocatalytic activity can be effectively enhanced by doping the material with foreign ions, which may be metals, non-metals^22,23^ and rare earth metals^24^. Changing out the oxygen or Ti^+4^ atoms with foreign ions with comparable ionic radii allowed for this enhancement to happen. The actual band gap of TiO_2_ is subsequently reduced due to the introduction of new energy levels; furthermore, the foreign ions serve as charge trapping sites, further reducing recombination processes. The concentration and type of dopant determine the band gap narrowing that led to visible light absorption. Nevertheless, the effect of different types of external species in TiO_2_ lattice have been studied it would highly fascinating to study the consequence of doping a Se (rarely considered as a metalloid) that has not been extensively explored. Aside from removal, it is quite important to detect aromatic chemicals in water streams, which necessitates the creation of an economical and straightforward technique for this purpose. Many analytical and spectroscopic techniques, such as ion-gas chromatography, cyclic voltammetry, mass spectrometry, Raman spectroscopy, and ion mobility spectroscopy, have been used for sensing applications^25^. However, the complexity, expense, and longer response times of their assay procedures restrict their uses. Fluorescence spectroscopy, on the other hand, demonstrates a promising analytical method in terms of sensitivity, selectivity, and quick detection of organic pollutants. Based upon this insight and motivation of developing a material capable of eliminating as well as detection of organic pollutants we have developed a nanostructure between TiO_2_ and SeO_2_ using simple ultrasonic technique. The catalyst was fully characterized by PXRD, XPS, Raman Spectroscopy, SEM, TEM, BET and UV–Vis spectroscopy. Dual performance of TiO_2_–SeO_2_ nanostructure for visible light photocatalytic degradation of 2,4,6-trinitrophenols as well as a selective sensing platform of picric acid in the aqueous phase has not been explored earlier. The experimental investigations were supported by the detailed theoretical DFT calculations in order to understand the functional aspect of TiO_2_–SeO_2_ nanostructure. To best of our knowledge no study has been reported so far portraying this kind of work.

## Experimental details

### Synthesis and characterization of TiO_2_:SeO_2_ nanostructure

**Figure 1:**
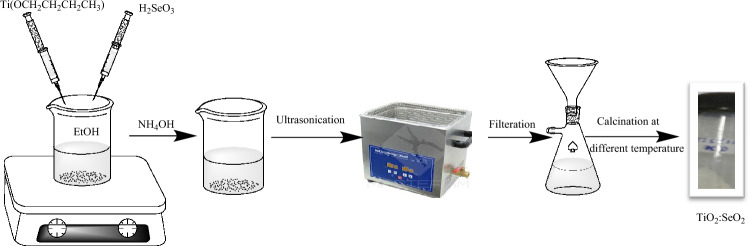
Schematic representation of TiO_2_:SeO_2_ nanostructure synthesis using ultrasonication method.

TiO_2_:SeO_2_ nanostructure was synthesized from titanium (IV) butoxide and selenous acid as a precursor of TiO_2_ and SeO_2_ respectively, with Ti:Se ratio of 1:0.1 (ST550). To achieve a homogeneous solution, titanium (IV) butoxide (3.4036 g) was dissolved in ethanol (20 mL) with vigorous and continuous stirring followed by addition of 10% NH_4_OH dropwise with constant stirring until complete precipitation occurred. The resulting reaction mixture was stirred for another half an hour before being sonicated in an ultrasonication bath for half an hour. Following that, an alcoholic solution of selenous acid (128.97 mg in 20 mL ethanol) was added dropwise with stirring. The resultant reaction mixture was eventually ultrasonicated for 40 min at a constant temperature (55 °C). The white suspension obtained was kept overnight without disturbing the solution and afterward filtered using a vacuum filtration assembly. The white precipitates of synthesized material were dried in an oven at 110 °C for 2 h and were subjected to additional calcination in a muffle furnace (for 2 h) at various temperatures, namely 350 °C, 450 °C, 550 °C, and 750 °C, and were labeled as ST350, ST450, ST550, and ST750, correspondingly. To optimize the synthesis conditions, TiO_2_:SeO_2_ nanostructures were prepared by varying Ti:Se ratios (1:0.1, 1:0.2) and solvent butanol, and synthesized nanostructures are assigned as ST550, ST2, and STB. Figure 1 illustrates the steps involved for synthesizing desired TiO_2_:SeO_2_ nanostructures.

X-ray diffraction analysis of TiO_2_:SeO_2_ nanostructures was done on the D8 ADVANCE Bruker model of diffractometer using Cu Kα radiation (λ = 1.5418 angstroms) at a scan rate of 10° min^−1^. The morphology studies were done using FESEM and TEM recorded on Carl Zeiss, Merlin compact model and TECNAI, 200kV TEM respectively. The XPS spectra of the nanostructureswere recorded in the spectrometer of the Nexsa base model using Al Kα radiation as the source. The spectral analysis i.e., FT-IR spectra were obtained on RZX, Perkin Elmer Spectrometer across a 4000–400 cm^−1^ range and at 4 cm^−1^ resolutions. The UV–Vis spectra and fluorescence spectra were recorded on a UV–Vis spectrophotometer (UV-1800, Shimadzu) and Spectrofluorometer (Shimadzu, RF-5301PC).

### Evaluation of photocatalytic potential of ST550

The photocatalytic activity of ST550 was carried out in a photocatalytic chamber using a 250 ml double-walled reaction vessel. The reaction mixture containing 2,4-dichlorophenol solution and ST550 was agitated in the dark to reach the state of adsorption–desorption equilibrium. The resulting solution was illuminated at different wavelengths of light (18 W) for 6 h in an oxygen-rich environment using an aerator pump with continuous magnetic stirring in the photocatalytic chamber. Samples were taken at regular intervals and filtered through a 0.22 µm syringe filter and the fluctuation in intensity (absorbance) was recorded using a UV–visible spectrophotometer (Shimadzu UV-1800) in order to track the degradation rate at wavelength 284 nm. The photocatalytic degradation efficiency (%) of 2,4-DCP was evaluated using the Eq. (1):

1 $${\text{Degradation}}\;{\text{efficiency }} = \, \left[ {{1} - \left( {{\text{A}}/{\text{Ao}}} \right)} \right] \, \times { 1}00$$

where Ao represents the starting concentration of 2,4-DCP and A represents the concentration of 2,4-DCP after exposure of visible light.

After photocatalysis, the utilized photocatalyst (ST550) was recovered, frequently washed with deionized water, and dried for 3 h at 110 °C. It was subsequently reused for five separate runs to evaluate the photocatalytic capabilities and reusability potential.

The mineralization of 2,4-DCP in terms of COD removal efficiency (%) of nanostructure ST550 was assessed against wastewater samples (effluent) collected from the paper and pulp industry in Sunam, Punjab (India), using multiparameter photometer with COD (Hanna instruments, HI839800). The % COD removal was assessed using the Eq. (2).

2 $$\% {\text{ COD}}\;{\text{ removal }} = \, \left[ {\left( {{\text{Co }}{-}{\text{ Ct}}} \right)/{\text{Co}}} \right] \, \times { 1}00$$

where, Co and Ct are COD in ppm of 2,4-DCP before and after time ‘t’.

### Radical scavenger (quencher) studies

Free radical trapping investigations were conducted for identifying the reactive species in the course of the photodegradation of 2,4-DCP using p-benzoquinone (BQ) as the superoxide radical (^**·**^O_2_^−^) scavenger, ammonium oxalate (AO) as the hole scavenger (H^+^) and isopropyl alcohol (ISP) as the hydroxyl radical (^**·**^OH) scavenger.

### Evaluation of the fluorescence potential of ST550 for sensing organic pollutants (OPs).

Fluorescence spectra of ST550 and ST550/OPs were recorded by mixing OPs solution (10^–4^ M) and ST550 suspension (50 mg L^−1^) with a 1:1 (v/v) ratio at the excitation wavelength of 360 nm and slit of excitation and emission of 1.5 nm. The quenching effect of these pollutants was inspected by monitoring the relative change in fluorescence intensity (I_o_/I) of ST550 in the presence of different OP solutions.

### Computational details

First-principle Density functional theory (DFT) as implemented in CASTEP (using DMol3 package (27) with GGA-DFT [37]) was employed to optimize the geometry of synthesized nanostructure ST550 and also to understand the fluorescence quenching and photocatalytic degradation of organic pollutant molecules in the presence of ST550 nanostructure. Calculations were performed at the Perdew-Burke-Ernzerhof (PBE) level of theory (using plane-wave basis set) with energy cut-off for wavefunction as 500 eV. The initial structures were made and relaxed until the force on each atom is < 5 × 10^–2^ eV/Å. The SCF convergence was set to be 2 × 10^–6^ eV/atom.

## Results and discussions

### Phase structure analysis of TiO_2_:SeO_2_ nanostructure

**Figure 2:**
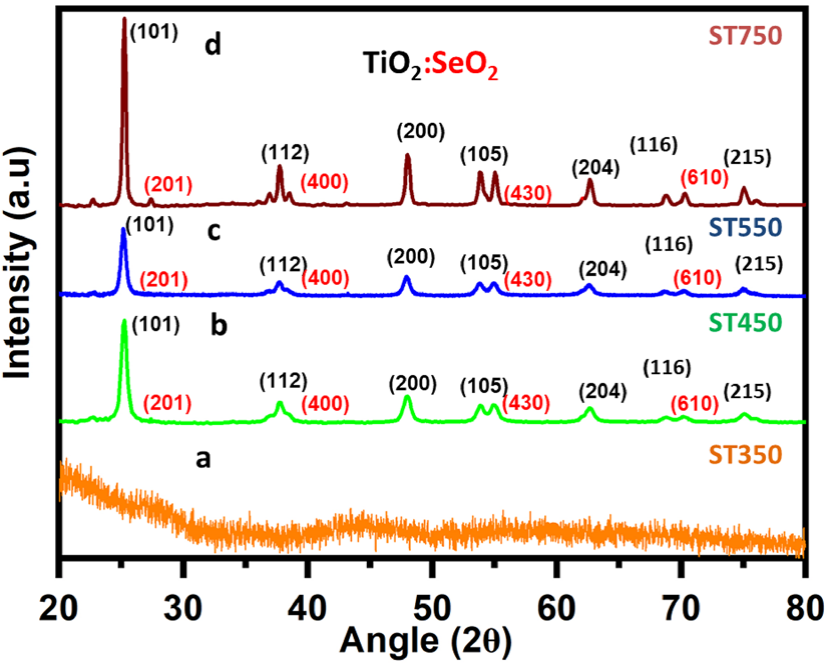
(a–d) PXRD spectra of ST350, ST450, ST550 and ST750.

The crystal structure analysis of the nanostructures ST350, ST450, ST550 and ST750 is done using PXRD analysis as shown in (Fig. 2a–d) below where we can see the prominent diffraction peaks positioned at 25.4°, 37.05°, 38.6°, 48.15°, 53.92°, 62.84°, 68.59° and 75.28° corresponding to planes (101), (112), (200), (105), (204), (116, (215) of anatase phase of TiO_2_ and the peaks positioned at 27.38°, 43.49°, 55.19°, and 68.84° corresponding to planes (201), (400), (430) and (610) of tetragonal phase of SeO_2_ validating the formation of nanostructure between SeO_2_ and TiO_2_.

Figure 2a illustrates the amorphous structure of ST350 due to the absence of distinct peaks for SeO_2_ and TiO_2_. Crystallization apparently happened at 450 °C and 550 °C, as can be seen from the well-defined peaks in the XRD of ST450 (Fig. 2b) and ST550 (Fig. 2c). The PXRD pattern of TiO_2_:SeO_2_ (ST2) with molar ratio 1:0.2 is shown in (Fig. S1). When the amount of SeO_2_ in the catalyst is increased, the intensity of the peaks corresponding to TiO_2_ decreases while the intensity of the peaks corresponding to SeO_2_ increases. The sample's XRD peaks are intense, indicating that the formed nanomaterials are crystalline, and broad diffraction patterns indicate a very small-sized crystalline structure. Furthermore, the increase in FWHM was noticed, pointing towards the decrease in size with an increase in the SeO_2_ content however lattice strain increases.

The size of the crystallite and strain on the lattice of photocatalysts that were calcined at various temperatures are shown in (Table S1). Scherer's equation and the lattice strain equation, respectively, were used to calculate the crystallite size and lattice strain values. For synthesized metal oxide nanomaterial, increasing the amount of SeO_2_ or decreasing the TiO_2_:SeO_2_ ratio resulted in a decrease in crystallite size and an increase in lattice strain. The addition of SeO_2_ reduced the orderly arrangement of particles, causing strain in the metal oxide lattice. ST550 and ST2 crystallite sizes and lattice strains were calculated to be 176.67 nm, 28.99 nm, and 0.0526, 0.3154, respectively.

Crystallite sizes of TiO_2_-based photocatalysts calcined at different temperatures ST450, ST550, and ST750, are found to be 90.64 nm, 176.67 nm, and 208.66 nm, respectively, and their calculated lattice strains are 0.1052, 0.0526, and 0.0449, respectively indicating that crystalline size increases with increasing calcination temperature whereas lattice strain decreases. All the photocatalysts synthesized have crystallite sizes in the range of 28–210 nm.

### Morphological analysis of TiO_2_:SeO_2_ nanostructures

**Figure 3:**
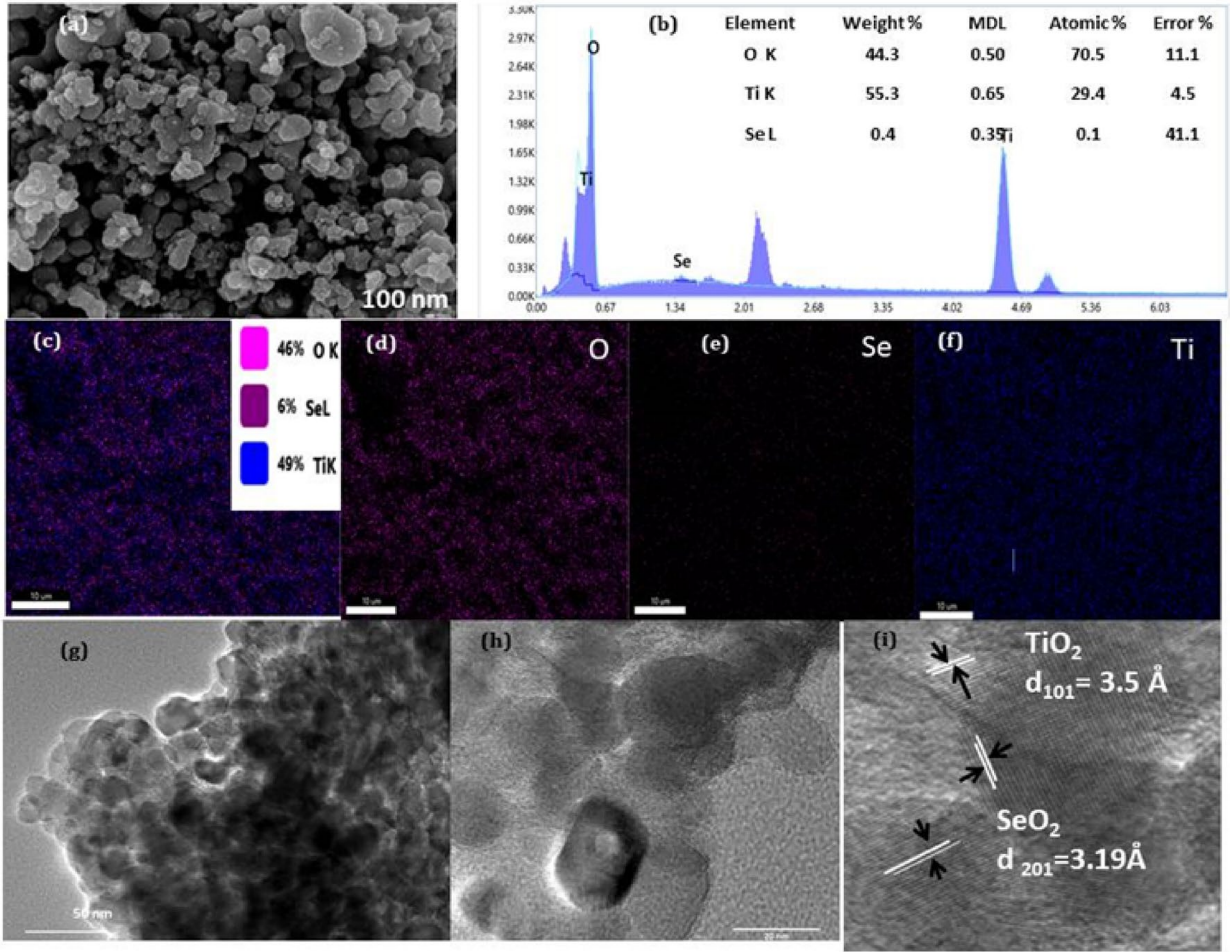
(**a**) SEM image, (**b**) EDX spectra with elemental percentage, (**c**–**f**) Elemental mapping of ST550 (**g**) TEM image of ST550 (**h**, **i**) HRTEM image of ST550.

Scanning electron microscopy and transmission electron microscopy were used to investigate the morphological properties of TiO_2_:SeO_2_ nanostructures. The morphological analysis was carried out on calcinated TiO_2_:SeO_2_ nanostructures (ST350, ST750 and ST2), and SEM images of ST350 and ST750 are shown in Fig. S2 and SEM image of ST2 along with EDX spectra is shown in Fig. S3. The SEM image of ST550 revealed the formation of uniform globular nanoparticles (Fig. 3a) and the incorporation of SeO_2_ into TiO_2_ was confirmed from the EDX spectra (Fig. 3b) and elemental mapping Fig. 3c–f. It is well known that as the calcination temperature rises, the size of nanoparticles increases, and at 750 °C, particles get clustered^26^. Chemical microanalysis (employing EDX) was used to determine the elemental composition, which confirmed the increase in Se content parallel to Ti:Se ratios. The weight % of Se for ST550 and ST2 were found to be 0.97, and 1.98, respectively authenticating the addition of Se. A subsequent TEM image of ST550 (Fig. 3g) also confirms the formation of globular nanoparticles. The high resolution TEM image (Fig. 3h,i) indicates the appearance of lattice fringes with d spacing 3.5 Å and 3.19 Å respectively which further corresponds to (101) plane of TiO_2_ and (210) plane of SeO_2_. The SEM images of TiO_2_:SeO_2_ photocatalysts solvents (Fig. S4), including ethanol and butanol, reveal that changing the solvent had a noticeable effect on the size and morphology of the TiO_2_:SeO_2_ nanostructures. This pattern can be explained by high solvent viscosity, surface tension, and higher molecular weight, which inhibited cavitation due to higher natural cohesive forces acting within the liquid^27^.

### Spectral investigation of TiO_2_:SeO_2_ nanostructures

#### XPS spectral analysis

**Figure 4:**
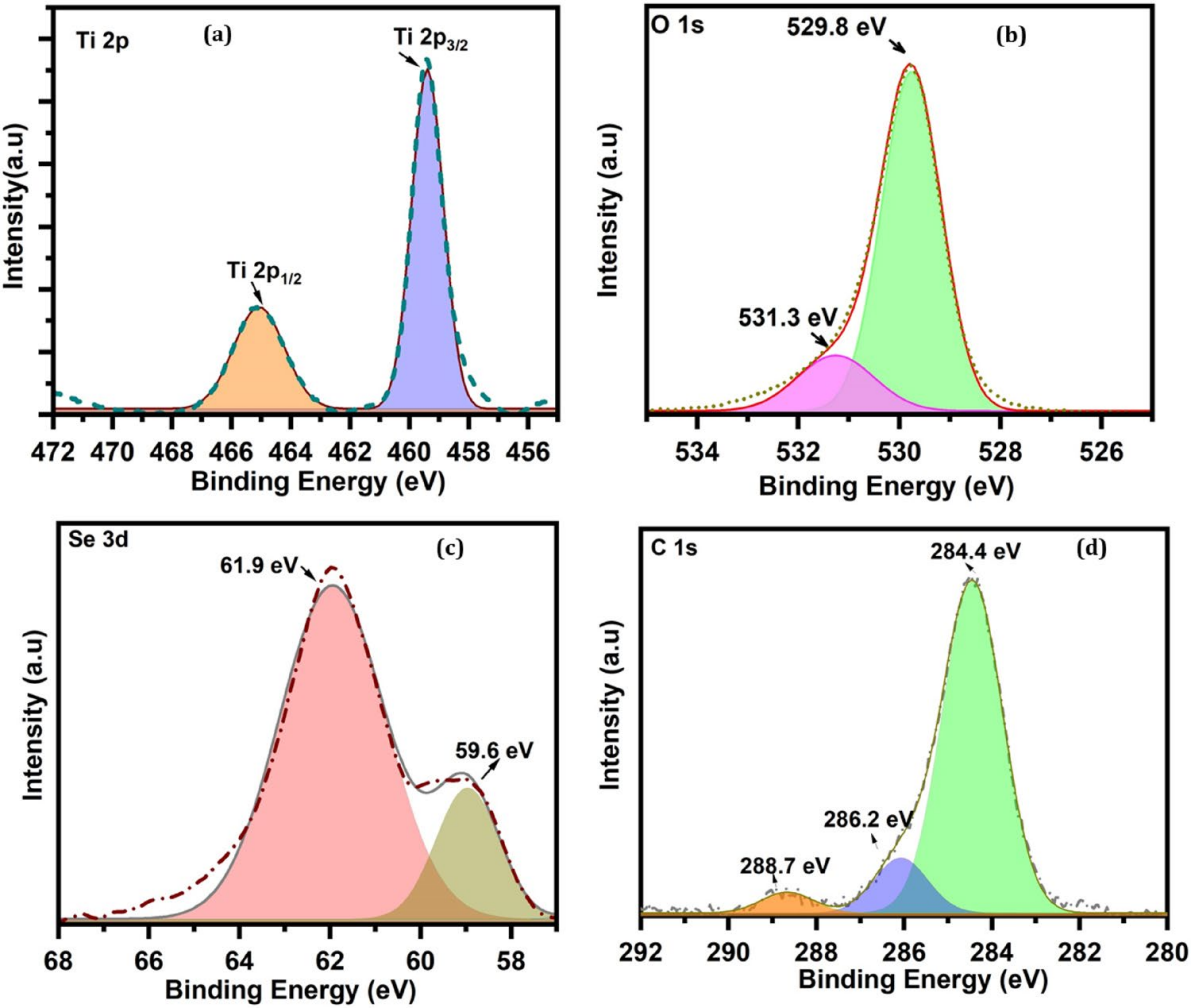
High resolution XPS spectra of ST550: (**a**) Ti 2*p* (**b**) O 1*s* (**c**) Se 3*d* (**d**) C 1*s*.

Further, structural elucidation of TiO_2_:SeO_2_ nanostructures were augmented by spectral investigations such as XPS, Raman, FTIR, UV–Vis, and Fluorescence. The electronic structure and chemical states of Se-doped TiO_2_ were investigated using XPS. The wide scan survey spectra of ST550 contains peaks which can designated to Ti 2*p*, C 1*s*, Se 3*d* and O 1*s* confirming the formation of heterostructure between TiO_2_ and SeO_2_ as shown in (Fig. S5). The Ti 2*p* peak (Fig. 4a) is deconvoluted into two peaks at 458.9 eV and 464.5 eV that confirms the Ti’s primary valence state in ST-550 is + 4. The O 1*s* peak in (Fig. 4b) can be deconvoluted into two peaks at 529.9 eV and 531.3 eV. The peak at 529.9 eV signifies the lattice oxygen presence in TiO_2_ and the peak at 531.3 eV can be dedicated to lattice oxygen presence in SeO_2_^28^. The Se 3*d* XPS peak is depicted in (Fig. 4c), which can be deconvoluted into two peaks at 61.9 eV and 59.6 eV respectively. The peak at 59.6 eV is responsible for Se^+4^ whereas adjacent peak at 61.9 eV is credited to the binding energy of Ti 3*s*^29,30^. The C 1*s* peak in (Fig. 4d) can be discretized into a sharp peak at 285.16 eV. Carbon peak is endorsed to the residual carbon from the material and hydrocarbon from XPS instrument itself. Because of significant lattice distortion in the ST-550 with 2.26 atomic % Se concentrations, the possibility of Se occupying the lattice sites is ruled out. Because of the formation of the Ti–O–Se structure, oxygen may attract electron density from Se ions in addition to nearby Ti ions.

#### Raman spectral analysis

**Figure 5:**
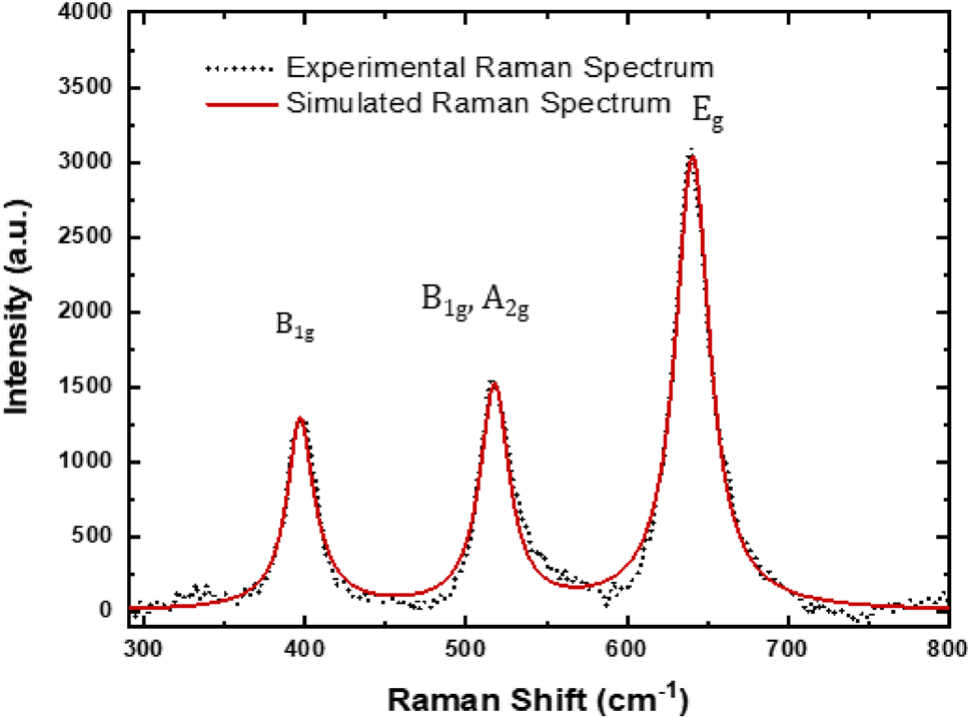
Experimental and PCM simulated Raman Spectrum of ST550.

The experimental Raman spectra is given in (Fig. 5) show peaks corresponding to 398.64 (B_1g_), 518.38 (B_1g_, A_2g_), and 639.89 (E_g_) cm^−1^^31^ confirming the formation of TiO_2_ nanomaterial. Theoretical Raman studies were also carried out by the phonon confinement model as described in our previous work^32^. Considering the Gaussian-shaped envelope function and imposing the limit of finite-sized crystal structure, the following equation can be written,

3 $$I\left(\omega \right)=4\pi \sum_{i=1}^{n}{\text{A}}_{(\text{i})}{\int }_{0}^{\pi /a}\frac{{q}^{2}\text{exp}(-{q}^{2}{d}^{2}/2\alpha )}{{[\omega -{\omega }_{o\left(i\right)}-{\gamma }_{\left(i\right)}\{1-\text{cos}\left(qa\right)\}]}^{2}+{({\Gamma }_{o(i)}/2)}^{2}}dq$$

where, q is the reciprocal space coordinate, Γ_0_ is the natural line-width, and A_(i)_ is the weight-factor for contribution of phonon (located at ith zone-center) to the Raman scattering with first order. The simulated Raman spectrum is then obtained by solving Eq. (3) by integration over the entire zone and plotting the value of I(ω) against ω. The results obtained are presented in Table 1. It was found that this model could simulate the spectrum very well and the spectral parameters were evaluated as given in Table 1.

**Table 1:** Raman spectral parameters evaluated by simulation using phonon confinement model.

Peak position (cm^−1^)	Natural line-width (cm^−1^)
396.2	18
400.2	35
517.0	22
640.0	26

### FTIR spectral analysis

**Figure 6:**
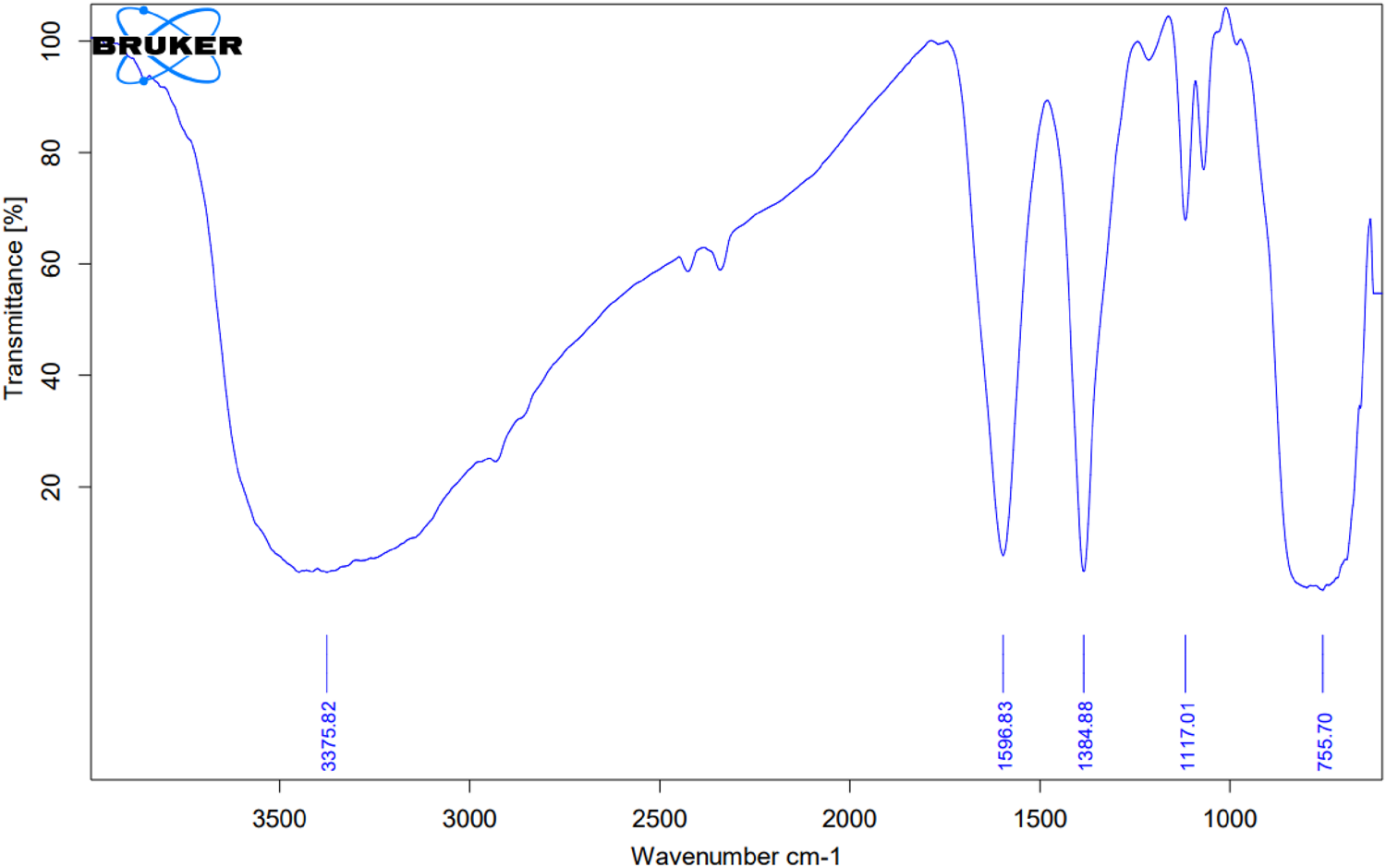
FTIR spectrum of ST550.

FTIR spectra (Fig. 6) reveal a strong broadband at 3376 cm^−1^, which corresponds to hydroxyl stretching. The peaks close to 1600 cm^−1^ and 1385 cm^−1^, respectively, were identified as the stretching vibration of the KBr bond and the vibration caused by the bending of H–O–H bond for water. A broad band peak with low intensity appears for the stretching selenium-oxygen vibration near 1250 cm^−1^ for the high selenium content (0.1 mol%). The narrow band near 800–700 cm^−1^ range has been attributed to vibrations from stretching of the Se–O bond and the Ti–O bond.

#### UV-Vis spectral analysis

**Figure 7:**
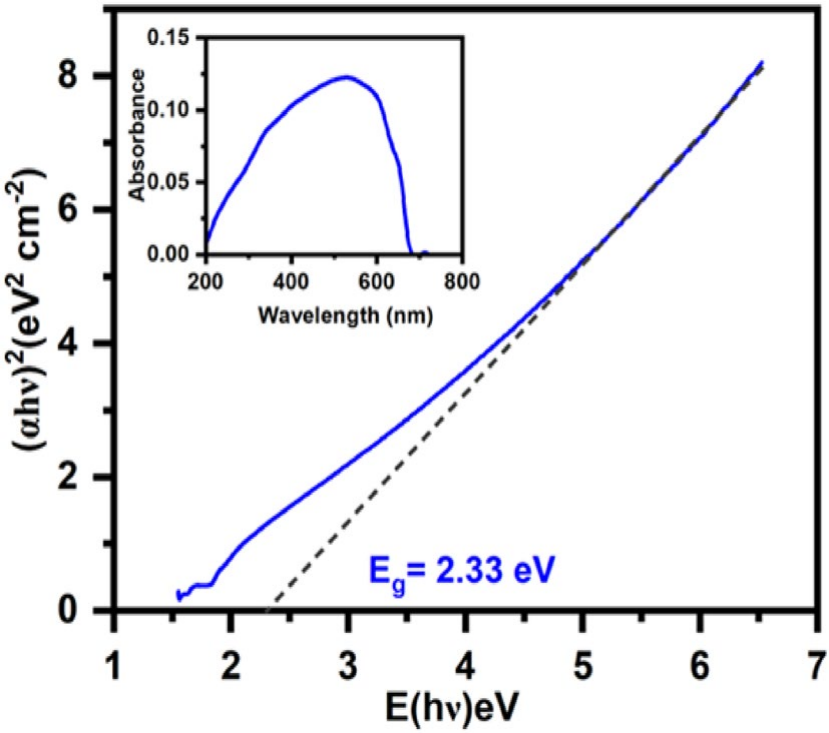
UV–Vis spectra and Tauc’s plot of ST550.

Band gap investigations of TiO_2_:SeO_2_ photocatalysts, calcined at diverse temperatures (from 350 to 750 °C) were performed by recording UV–Vis spectra and were shown in (Fig. S6). The UV–Vis spectra of ST550 (Fig. 7) showed broad absorption band with highly intense peak at 473.5 nm and band gap was calculated using Tauc’s plot. The comparison of band gap values obtained in case of other photocatalysts is shown in Table 2

**Table 2:** Comparison table of the band gap for ST350, ST450, ST750, ST2 and STB.

Photocatalysts	Band gap (eV)
ST350	5.29
ST450	3.39
ST550	2.33
ST750	5.74
ST2	2.39
STB	5.29

#### Fluorescence spectral analysis

**Figure 8:**
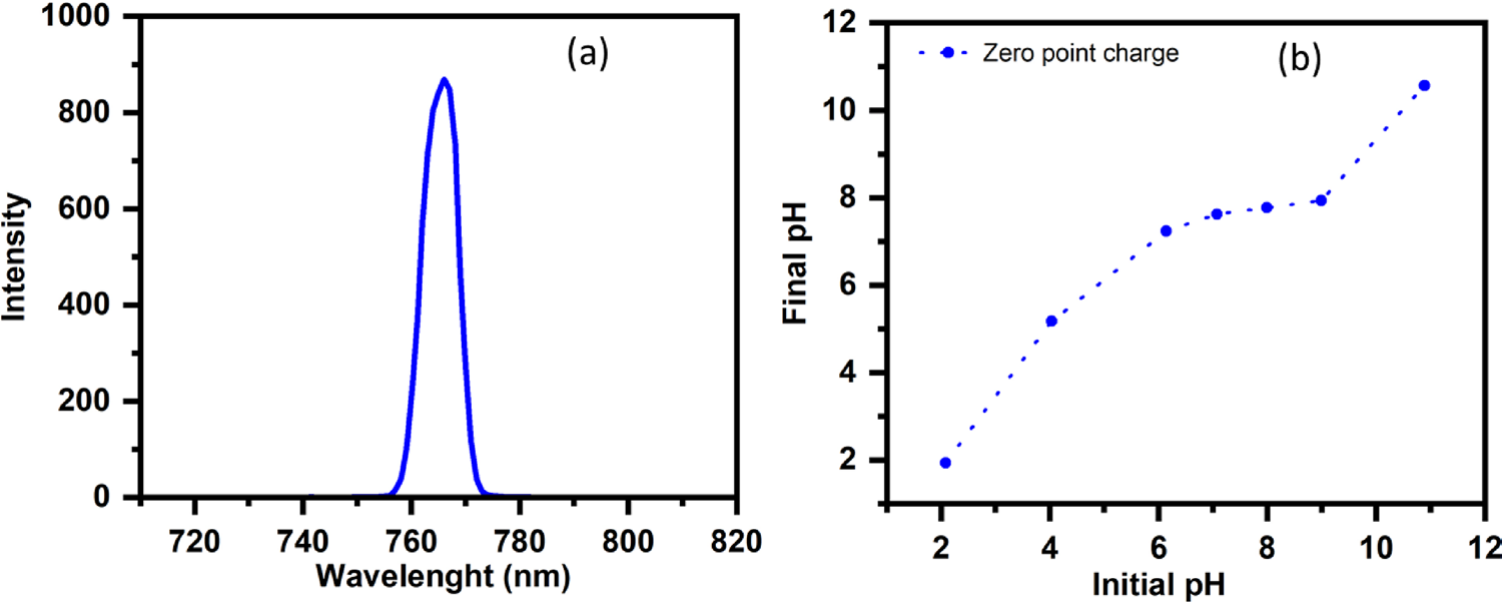
(**a**, **b**) Fluorescence spectra of ST550 and Zero-point charge (ZPC) of ST550.

The fluorescence qualities of the material are connected to the electronic structure, the fluorescence spectra reveal information on the degree of transfer and separation of photogenerated electrons and holes on the surface of photocatalysts upon excitation. The photoluminescence (PL) spectra of ST550 (Fig. 8a) revealed a prominent PL emission band positioned at 767 nm, with excitation wavelength 360 nm, which illustrates the fluorescent activity of the synthesized nanostructures (ST550).

#### Zero-point charge and surface area analysis

**Figure 9:**
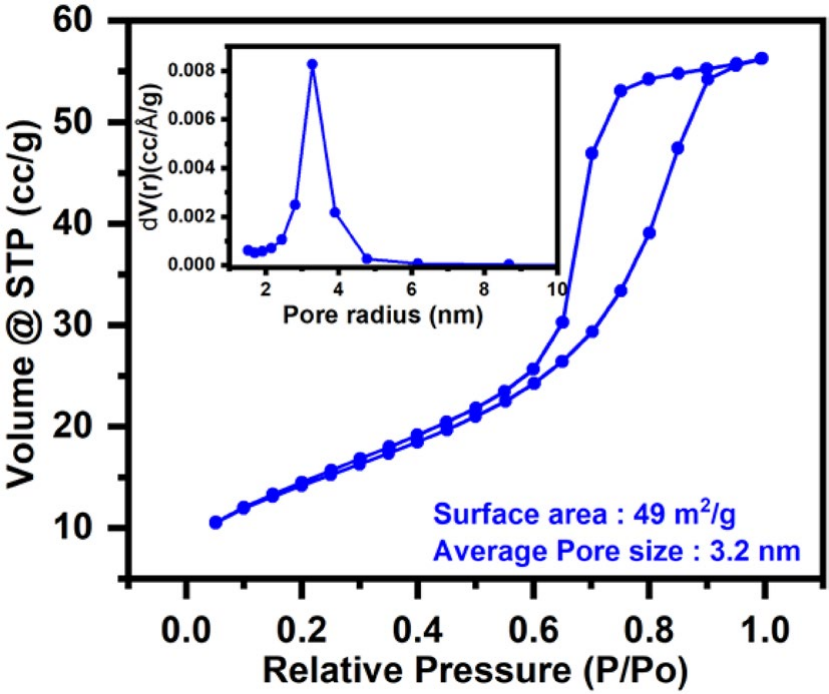
N_2_ adsorption/desorption and BJH pore size distribution curve (inset plot) of ST550.

The pH drift method was used to calculate the zero-point charge (ZPC) of ST550^33^ and ZPC for ST550 was found to be 7.54. (Fig. 8b). As a result of H^+^ ion adsorption on the surface of the catalyst, the catalytic surface is positively charged at pH less than 7.54. Above pH 7.54, however, adsorption of OH^−^ ions on the surface of the catalyst occurs, resulting in a negative charge on the catalyst. The nanomaterial's adsorption plays a significant role in influencing its catalytic effectiveness both as a photocatalyst and a fluorophore (majorly static quenching). Surface area analysis of the nanostructures was done using BET. The BET adsorption isotherm of ST550 is shown in (Fig. 9) along with BJH pore size distribution curve in the inset. ST550 has surface area of 49.0 m^2^ g^−1^ with average pore size 3.2 nm confirming the mesoporous nature of the ST550.

The BET isotherms of remaining nanostructures ST350, ST450, ST750, ST2 and STB are displayed in (Fig. S7) and a comparison of the parameters is provided in Table 3. It can be witnessed from the data provided in Table 3 that among all the nanostructures ST550 has more surface area leading to better adsorption of organic pollutants aiding the fluorescence quenching and improved degradation efficiency.

**Table 3:** Parameters extracted from the BET surface study of TiO_2_:SeO_2_ nanostrucutre.

Photocatalysts	Surface area (m^2^ g^−1^)	Pore size (nm)
ST350	33.96	4.7
ST450	47.0	3.9
ST550	49.0	3.2
ST2	18.3	1.6
STb	28.63	2.4

## Photocatalytic performance of TiO_2_:SeO_2_ nanostructures

**Figure 10:**
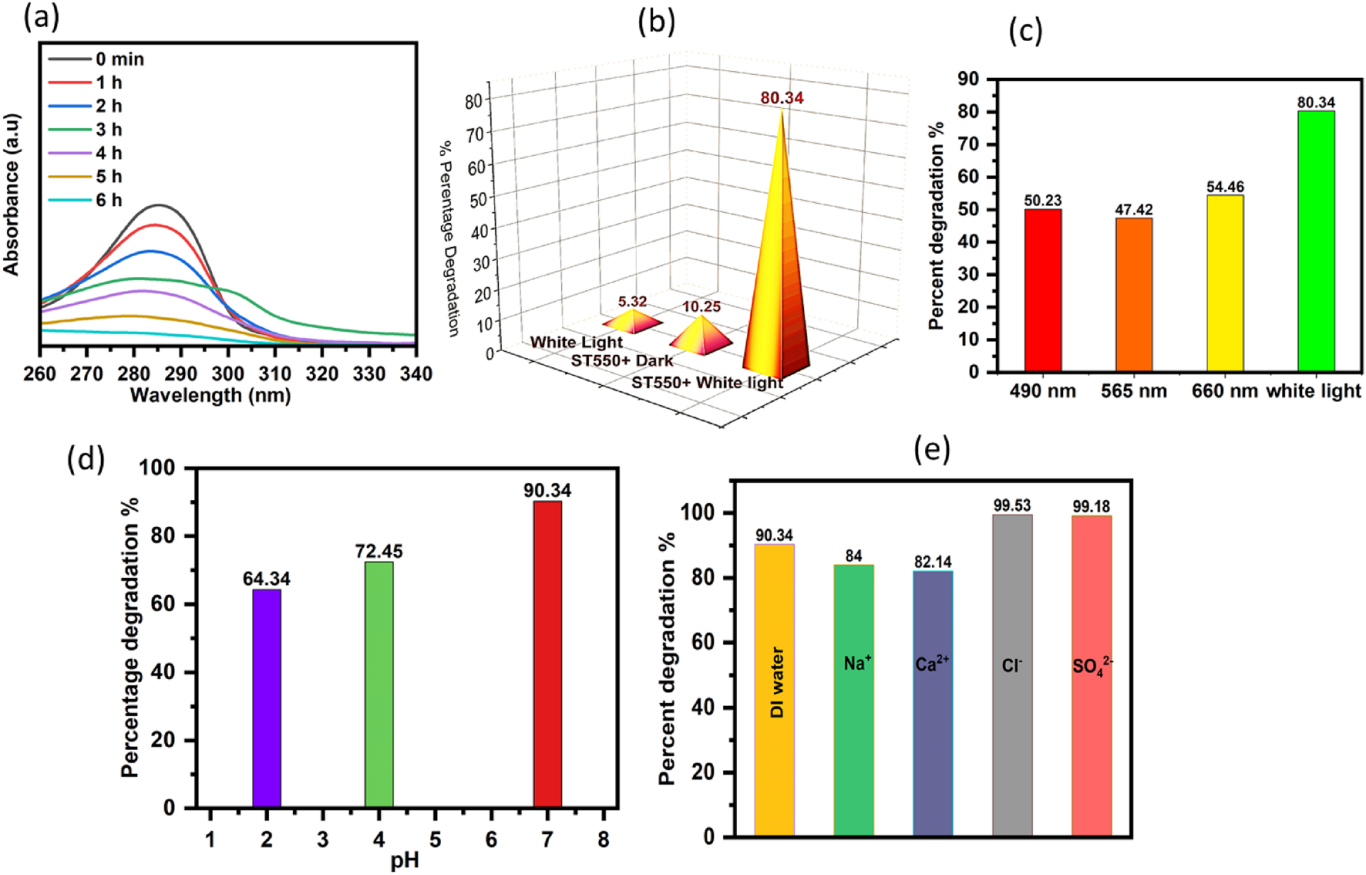
(**a**–**d**) Photocatalytic degradation of 2,4-dicholophenol was carried out using synthesized photocatalyst ST550 (**a**) UV–Vis time dependent spectra under optimized reaction parameters, (**b**) Photocatalytic degradation under different conditions, (**c**) Effect of wavelength on photocatalytic degradation, (**d**) Effect of pH on photocatalytic degradation and (**e**) Influence of various inorganic ions during the photocatalytic degardation.

The photocatalytic performance of the synthesized photocatalysts for the degradation of the organic pollutant 2,4-DCP was evaluated (a phenolic derivative). The photocatalytic decomposition of 2,4-DCP was carried out utilizing a synthesized ST550 under numerous wavelengths of light by altering the reactions parameters such as the concentration and pH of the model compound, catalyst concentration, and presence of different cations and anions in the solution. The rate of deterioration was calculated using the rate of a shift in the absorbance with time at the model compound's wavelength maxima. The UV–Vis spectra of 2,4-DCP revealed a characteristic absorption peak in the UV region at 284 nm. (Fig. 10a**)** depicts the time-dependent UV–Vis spectra of 2,4-DCP throughout photocatalytic degradation using white light. The intensity of absorption decreases with irradiation time and it was calculated that 90.34% of the 2,4-DCP was degraded in 6 h using the ST550 photocatalyst under optimized reaction parameters.

Furthermore, to analyze the contribution of adsorption and photolysis under light, the photocatalytic degradation of 2,4-DCP was examined under various conditions (white light, ST550/dark, ST550/white light) (Fig. 10b). It was observed that when a blank study was carried out in the absence of nanostructured ST550, only 5.32% degradation occurred, while 10.25% degradation occurred for ST550/dark conditions. Finally, under ST550/light conditions, the degradation of 2,4-DCP was found to be 80.34%. The photocatalytic response of ST550 to the degradation of 2,4-DCP was investigated using LEDs with different visible wavelengths (blue, green, red, and white light) as shown in (Fig. 10c). The degradation efficiencies of 2,4-DCP for blue, green, red, and white light were 50.23, 47.41, 54.46, and 80.34%, respectively. The percentage degradation was found to be greatest for the sample exposed to white LED with wavelength (390–700 nm), so further research was conducted with white LED. The percentage degradation of 2,4-DCP was measured at pH ranges ranging from 2 to 7, as shown in (Fig. 10d), to look at the impact of pH on ST550 degradation efficiency. The percentage decay was discovered to be greatest at pH 4. At pH less than 7, the surface of the ST550 catalyst is positively charged, whereas the surface of the pollutant is negatively charged due to a lone pair of electrons on chlorine atoms, resulting in better ionic attraction between both surfaces and thus higher degradation.

Industrial wastewater comprises a variety of inorganic cations and anions, so it is critical to investigate how these ions affect the photocatalytic breakdown of 2,4-DCP using ST550. Figure 10e depicts the effect of various cations and anions on the percentage degradation of 2,4-DCP. These ions could be isolated or included in ionic complexes^34^. The active radicals produced during photocatalysis can easily interact with free ions, transforming them into ionic radical species which take part in the photocatalytic activity^35^. The ST550 function mostly unchanged in the presence of anions. However, adding cations to the solution lowers the rate of deterioration. This finding emphasizes how cations block the 2,4-DCP ability to interact with ST550 photocatalyst^35–37^. The degradation effectiveness declines in the following order: SO_4_^2−^ > Cl^-^ > Na^+^ > Ca^2+^.

**Figure 11:**
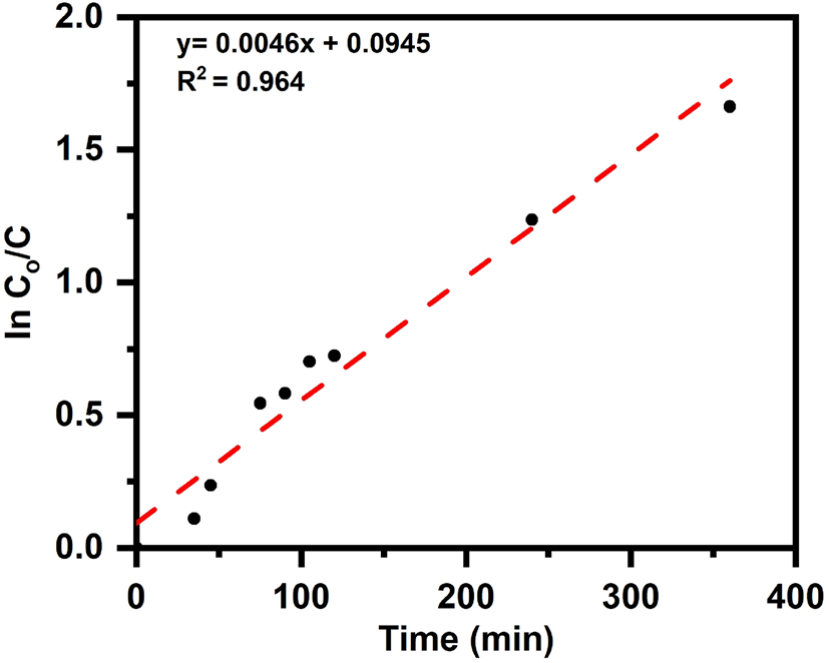
Kinetic study of 2,4-Dichlorophenol under optimized conditions (Catalyst dose—1 g/L, pH-7, initial concentration—10 ppm).

Degradation analysis under optimized reaction parameters such as catalyst dose of 1 g/L, pH of the 2,4-DCP solution is 7, and initial concentration of 2,4-DCP was 10 ppm were performed to determine the disappearance time of 2,4-dichlorophenol (10 ppm) by altering the duration range 5 to 350 min. The results revealed (Fig. 11) that the apparent first-order kinetic model was followed throughout the photocatalytic degradation of 2,4-dichlorophenol by utilizing ST550, with a rate constant (K) of 0.0046 min^-1^ and a correlation constant (R) of 0.964.

The performance of the nanostructure is compared with other photocatalysts reported in the literature as shown in Table 4. From the parameters mentioned in the data it can clearly visualized that synthesized nanostructure is showing better performance from the reported ones.

**Table 4:** Comparison of the performance of the nanostructure with other photocatalysts reported in literature.

Photocatalyst	Synthesis method	Targeted pollutant	Light irradiation	Efficiency (%)	Reference
Mn/Mg–CoFe_2_O_4_–CaCr_2_O_4_–Ag_3_PO_4_ QDs	Sonochemical	Cefixime	Visible-light	99.8	^38^
SnS_2_/CPVC nanocomposites	Hydrothermal	Cr(VI) reduction	Visible-light	90	^39^
CQDs/NiFe_2_O_4_co-decorated MoO_3_-x/CoCr_2_O_4_ composite	Hydrothermal	DOXY	Visible-light	99.97	^40^
Au@Mg nanospheres	Simple mixing	Polystyrene	Halogen lamp	98.7	^41^
Fe_3_O_4_/FeWO_4_	Hydrothermal	Cr(VI) reduction	Xe lamp	99.0	^42^
CeO/g-C N	Wet-chemical	2,4-DCP	Visible-light	57	^43^
TiO/g-C N	Hydrothermal	2,4-DCP	Visible-light	46	^44^
TiO modified ZnO/SnO	Co-precipitation	2,4-DCP	UV light	72	^45^
CdTe/CdS, CdTe/CdS/N-rGO	Solvothermal	2,4-DCP	Visible light UV light	70 90	^46^
NaBiS_2_	Hydrothermal	2,4-DCP	Visible light	86	^47^
TiO_2_:SeO_2_	Hydrothermal	2,4-DCP	Visible light	90.34%	This work

**Figure 12:**
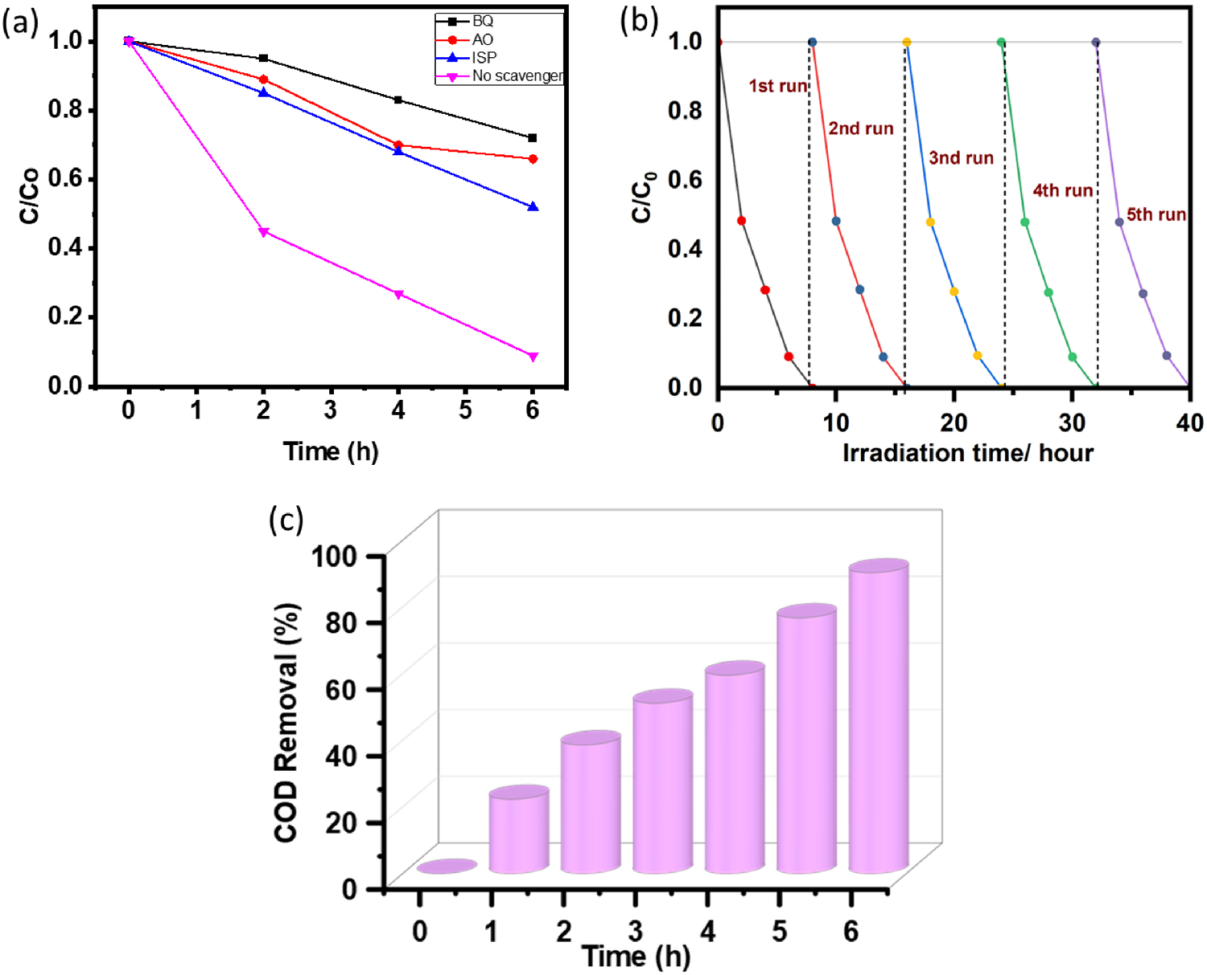
(**a**–**c**) (**a**) Effect of different quencher on degradation of 2,4-DCP, (**b**) Reusability of ST550 photocatalyst, (**c**) % COD removal of industrial effluent of paper and pulp mill.

Various radical scavengers to trap a series of active species in order to confirm which active species are involved in the photocatalysis process over the materials we prepared. The results of trapping experiment clearly suggest that h^+^, ^·^O_2_^−^, and ^·^OH all have significant effects on the photocatalytic performance (Fig. 12a). In comparison to the 90.34% degradation when no trapping scavenger is used, the rates of degradation drop to 35.84% and 48.30% when ammonium oxalate and isopropanol (1mL of 0.02 mM) are added, respectively. This indicates that both h^+^ and ^·^OH function as functioning species and have an impact on the photocatalysis of 2,4-DCP, with h^+^ having a greater impact on the degradation system than ^·^OH^44^. It is noteworthy that the 27.53% degradation rate is significantly reduced upon the addition of p- benzoquinone (1 mL, 0.02 mM), suggesting that ^·^O_2_^−^ plays a significant role in the photodegradation of 2,4-DCP. In conclusion, all three species h^+^, ^·^OH, and ^·^O_2_^−^ function well, with the resultant order of active radical species being ^·^O_2_^−^, > h^+^  > ^·^OH. The generation of ^·^OH radicals and degradation of 2,4-DCP can be described by the following Eqs. (4–8)

4 $${\text{ST}}550 + {\text{hv}} \to {\text{h}}^{ + } + {\text{e}}^{ - }$$

5 $${\text{H}}_{2} {\text{O}} + {\text{h}}^{ + } \to \;^{ \cdot } {\text{OH}} + \;^{ + } {\text{H}}$$

6 $${\text{O}}_{2} + {\text{e}}^{ - } \to \;^{ \cdot } {\text{O}}_{2}^{ - } $$

7 $$2^{ \cdot } {\text{O}}_{2}^{ - } + 2{\text{H}}^{ + } \to {\text{O}}_{2} + {\text{H}}_{2} {\text{O}}_{2}$$

8 $${\text{H}}_{2} {\text{O}}_{2} \to 2{\text{OH}}$$

The stability of the ST550 nanostructure during four consecutive deterioration cycles were investigated. After every usage, the catalyst was washed off with ethanol followed by Millipore water and then dried in a vacuum oven at 70 °C. The nanostructure maintained outstanding stability during all four rounds. Despite constant usage and washing, the catalyst's properties remained effective, in the fifth cycle, nanostructure successfully eliminated 89.9% of 2,4-DCP (Fig. 12b). The results demonstrated the catalyst's potential for cost-effectiveness and sustainability, and thereby eliminates the need for regular replacement.

## Mineralization of paper and pulp mill effluent

To assess the application of the produced photocatalysts, mineralization investigations were conducted in waste water samples collected from paper mill industrial. First, the effluent was filtered via syringe filters and frozen at 6 °C. The effluent was spiked with 2,4-DCP (20 mg L^−1^), and the photocatalytic degradation as a function of percentage COD removal was measured at ideal circumstances using 3 mL of the effluent in the presence of ST550. After 6 h of illumination of visible light with ST550 nanostructure, COD reduction was observed (90.23%), which indicates the mineralization of the 2,4-DCP in wastewater sample (Fig. 12c).

## Evaluating the efficiency of TiO_2_–SeO_2_ nanostructure as fluorescence sensors for picric acid (2,4,6 trinitrophenol)

**Figure 13:**
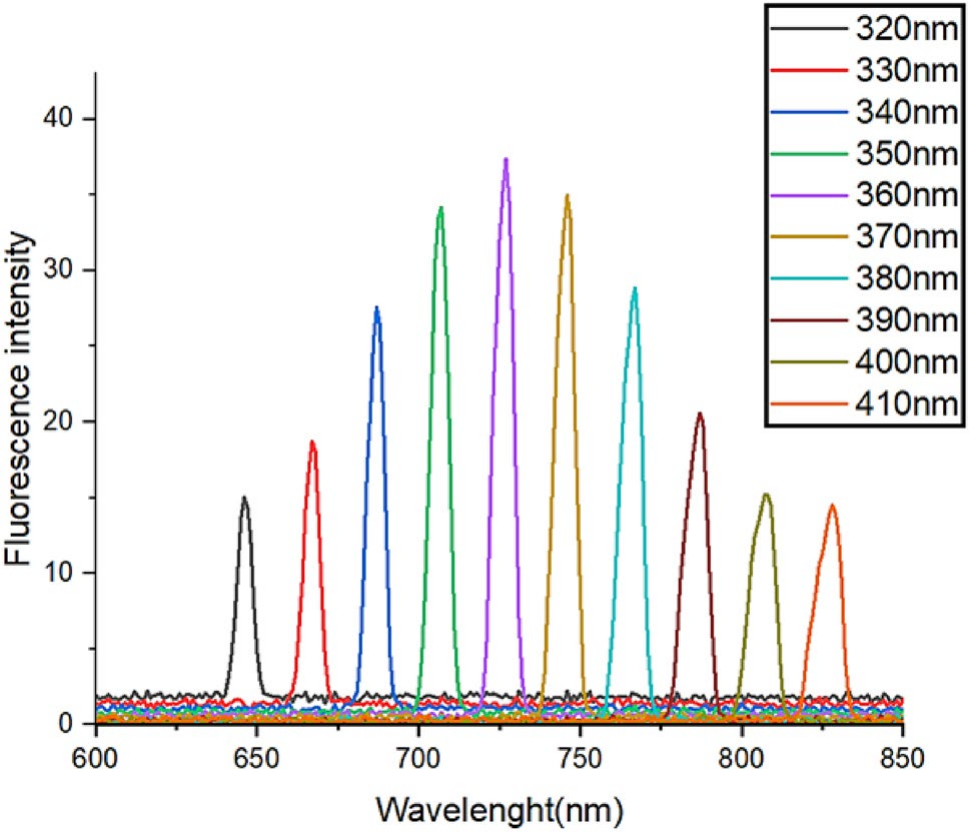
Fluorescence emission spectra of ST550 at different excited wavelengths (320 nm to 410 nm).

Photoluminescence spectra (PL) of ST550 nanostructure were also captured at various excitation wavelengths varying from 320 to 410 nm. According to the (Fig. 13), the fluorescence emission intensity of ST550 grew up to 360 nm and then started to decline.

**Figure 14:**
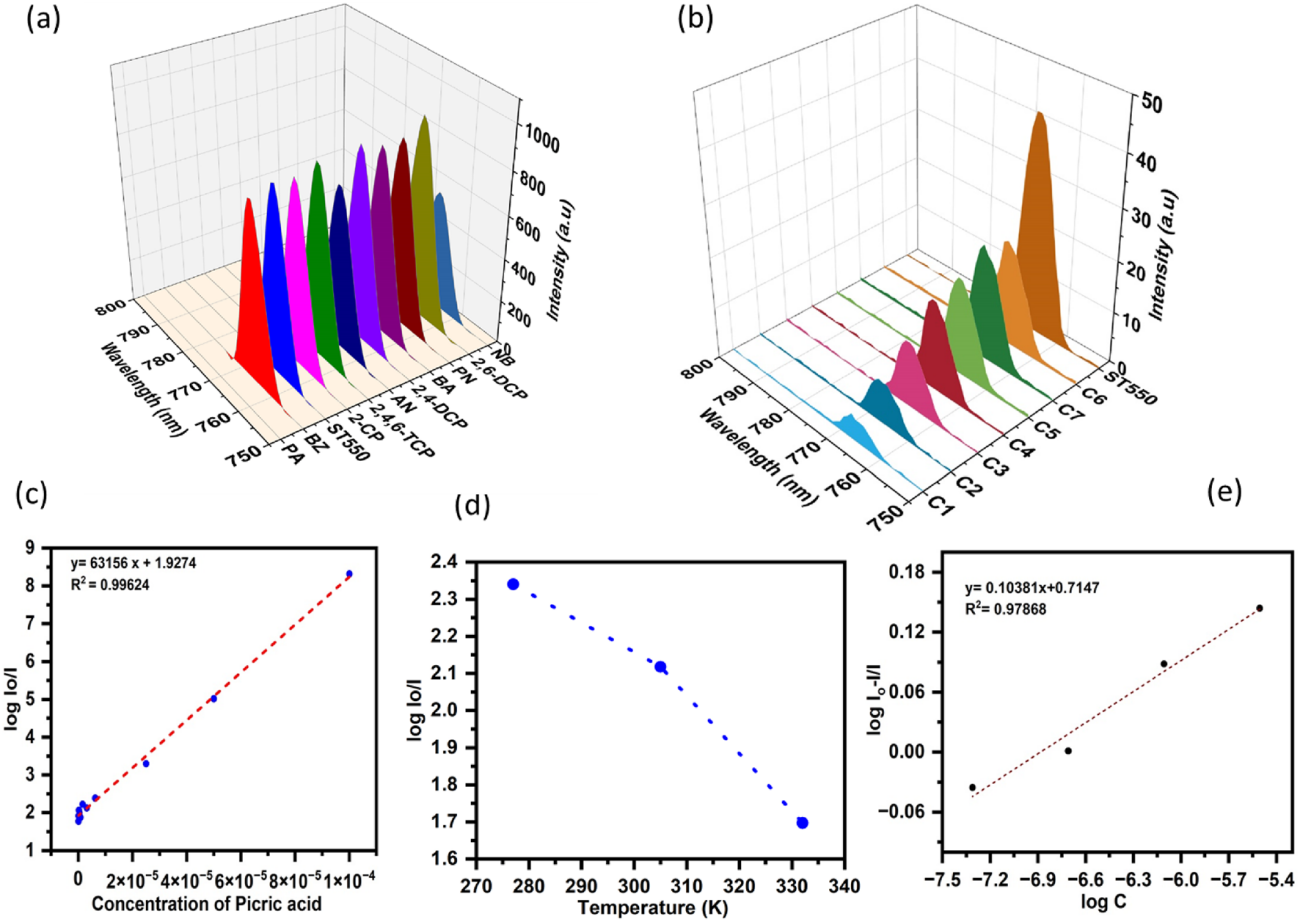
(**a**–**e**) ST550 photocatalyst as fluorescence sensors, (**a**) Fluorescence quenching ability of organic pollutants, (**b**) Effect of PA concentration on fluorescence quenching, (**c**) Stern–Volmer plot, (**d**) Effect of temperature on fluorescence quenching and (**e**) binding constant.

The PL spectra with excited wavelength 360 nm produced the highest fluorescence intensity hence, this wavelength was chosen as the optimized excitation wavelength for further fluorescence investigation. The fluorescence investigation for the efficiency of TiO_2_–SeO_2_ as a selective and sensitive fluorophore for PA was performed. The fluorescence ability of different aromatic organic pollutants such as benzaldehyde (BZ), 2-chlorophenol (2-CP), 2,4,6-trichlorophenol (TCP), aniline (AN), picric acid (PA), 2,4-dichlorophenol (2,4-DCP), benzoic acid (BA), phenol (PN), 2,6-dichlorophenol (2,6-DCP), and nitrobenzene (NB) was observed by tracking the shift in the ST550's relative fluorescence intensity as shown in (Fig. 14a). Among the different aromatic compounds, it has been observed that only PA shows a selective quenching of fluorescence emission of ST550 as evident from the variation in intensity of fluorescence spectra. A big aspect of the present work is the examination of the ST550 nanostructure's sensitivity for the detection of PA therefore, the influence of PA concentration on the fluorescence intensity of fluorophore (ST550) was investigated. The ST550 exhibits the highest level of fluorescence emission peak at 767 nm with excited wavelength 360 nm in the absence of PA and with an increase in PA concentration from 10^–4^ M to 7.8 × 10^–7^ M, the intensity of the emission peak gradually dropped (Fig. 14b), demonstrating a significant fluorophore interaction between ST550 and PA. Interestingly, over 95% quenching was accomplished when the concentration of PA was 10^–4^ M. The quenching of fluorescence can occur through a variety of processes, including collision quenching caused by molecular interaction, molecular rearrangement, excited state reactions, ground state complex formation, and energy transfer. There are two types of quenching mechanisms. There are two types of quenching: static and dynamic^48^. The data was incorporated into the Stern–Volmer equation (Eq. 9) to better understand the ST550 quenching mechanism with PA

9 $$\text{Io}/\text{I}=1+{K}_{sv}\left[C\right]$$

where Io and I are the intensities of fluorescence of ST550 and PA with ST550, respectively. The Stern–Volmer quenching constant is denoted by Ksv, and the concentration of PA or quencher is denoted by [C]. The Stern–Volmer quenching constant, 6.31 × 10^–4^ M^−1^, was calculated from the slope and linear Stern–Volmer plot is indicative of single quenching mechanism (Fig. 14c). A significant K_sv_ value indicates that the quencher and fluorophore have formed a complex (ST550 and PA).

For complete understanding of the type of quenching (Fig. 14d), the fluorescence spectra of ST550 (50 mg L^−1^) in the presence of 7.8 × 10^–7^ M PA were recorded at different temperatures (277 K–332 K). The results demonstrate that fluorescence quenching rises with decreasing temperature and is attributed to the development of a complex between ST550 and PA. At 332 K, this weakly bound complex dissociates, causing decreased fluorescence quenching, which is indicative of static quenching mechanism. The binding constant (K) and number of binding sites (n) can be determined from Eq. 10

10 $$\text{log}\left[\left({I}_{o}/ {I} \right)\right] =\text{log}K+n\text{log}[C]$$

The plot of $\text{log}\left[\left({I}_{o}-I/ {I}\right)\right]$ versus log [C] for ST550 and picric acid is shown in (Fig. 14e). The binding constant (K) for ST550 was found to be is 5.1844 M^−1^.

**Figure 15:**
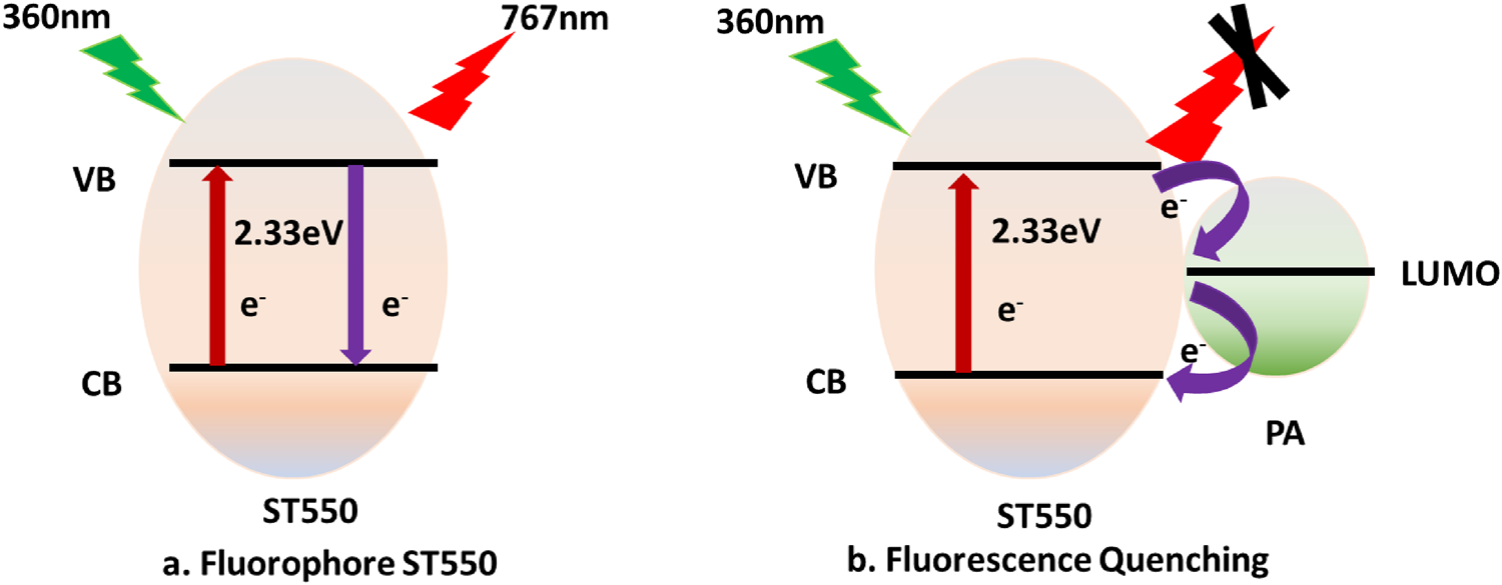
Plausible mechanism for fluorescence quenching of ST550.

An attempt has been made to explain the possible quenching mechanism (Fig. 15) of the fluorophore ST550 in the presence of quencher (PA). The energy gap for ST550 i.e., between CB (conduction band) and VB (Valence band) is 2.61 eV as established from spectral analysis using Tauc’s plot^2,29^. When light radiation of wavelength 360 nm (excitation wavelength) exposed on the surface of ST550 electron get transferred from CB to VB^49,50^. Thereafter, electron in the excited state undergoes several vibrational relaxation transitions, and finally de-excitation of electron to ground state results in emission of light with longer wavelength i.e., 767 nm (Fig. 15a). The molecules of PA get absorbed on the surface of fluorophore (ST550) and construct a complex ground state that facilitates the photo-induced electron transfer mechanism (PET), for the transfer of electrons. The presence of PA provides an additional energy level between the CB-VB of fluorophore (ST550), offering a supplementary pathway for electron de-excitation. The excited electron from ST550's LUMO is then transferred to the LUMO of PA, and eventually, it returns to ST550's ground state (HOMO), resulting in fluorescence quenching^48^ (Fig. 15b).

### Stability and recyclability of ST550 as a sensor

**Figure 16:**
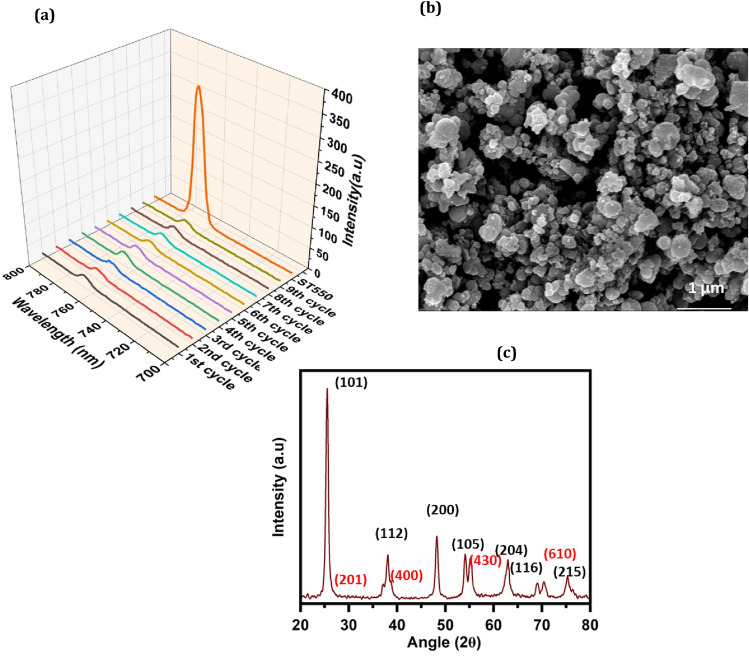
(**a**) Recyclability and stability of sensor, (**b**) SEM image, (**c**) PXRD spectra after recyclability.

Stability and recyclability of fluorophores are crucial in determining their reliability for utilisation. PXRD and FESEM were used to assess the stability of ST550 nanostructure after dispersing 40 mg of materials in 2,4-DCP for 48 h The recyclability of ST550 (fluoroprobe) was examined by reusing samples after sensing performance. Despite frequent use and washing, the fluorophore properties of ST550 remained effective; in the ninth cycle, nanostructure successfully sensed picric acid (Fig. 16a). PXRD study corroborated crystallinity and framework integrity after immersing in 2,4-DCP solution. FESEM images confirmed no change in surface morphology after sensing. (Fig. 16b,c).

## Theoretical investigations

The adhesion of molecules of organic matter on the surface of the ST550 nanostructure affects both the photocatalytic destruction of contaminants and the quenching of ST550. Theoretical calculations were performed as described in Section “Computation Details Radical scavenger (quencher) studies”. To investigate photocatalytic and fluorescence response at the molecular level, the ST550 has been used as substrate interacting with different organic moieties. The binding energy (B.E.) of specie (in present case, different organic pollutant molecules) with the substrate is calculated using Eq. 11

11 $$B.E. = E_{specie + substrate} - (E_{specie} + E_{substrate} )$$

As per the above equation, negative value of B.E. represents favorable interaction of species with the substrate. The optimized structure and corresponding energy of the TiO_2_:SeO_2_ nanostructure is given in Table 5.

**Table 5:**
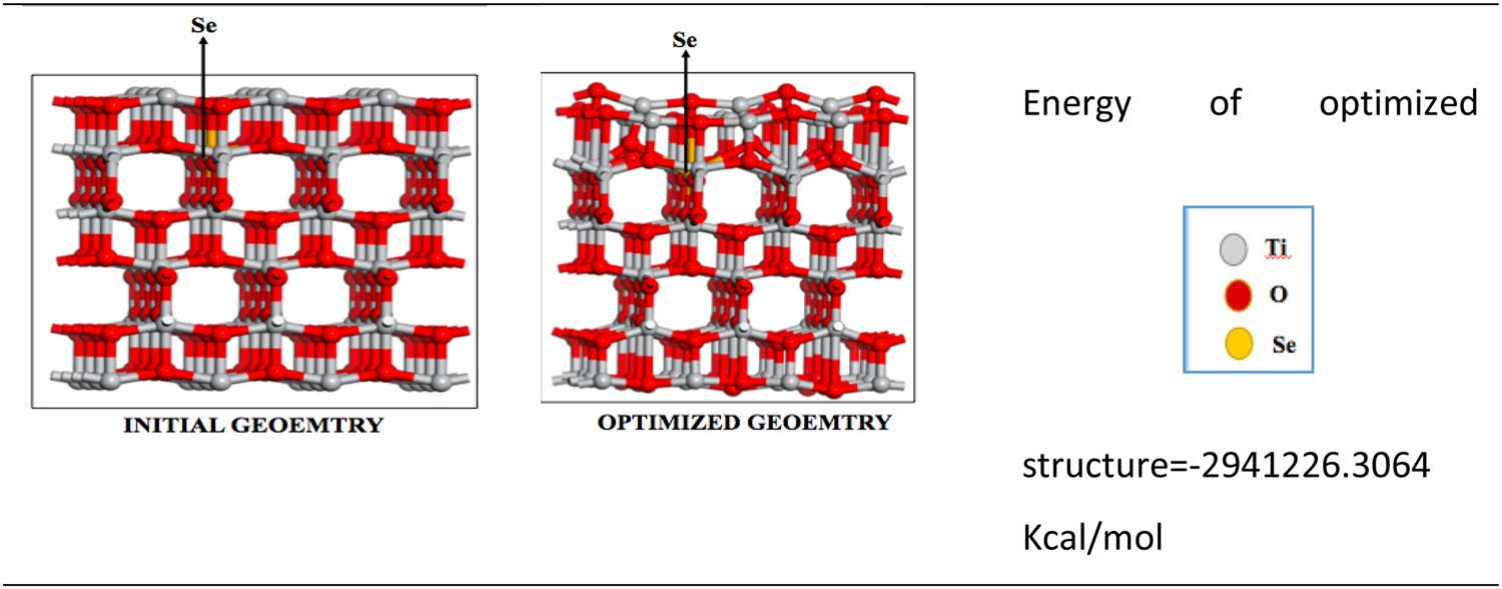
Initial and optimized geometry of the synthesized ST550 nanostructure.

Tables 6 and 7 list the optimized structure and energy of different adsorbing molecules as well as the adsorbed adduct of these molecules with ST550, respectively. On the basis of interactions of aromatic compounds with TiO_2_-SeO_2_, benzaldehyde and aniline showed a positive binding energy values + 15.98 and + 35.3 kcal/mol. This explained the experimental fact that these species do not show any fluorescence quenching effect on ST550. It was also found that the binding energy values for 2,4-DCP (− 12.31 kcal/mol) and PA (− 66.21 kcal/mol) with ST550 are significantly more negative and exhibits strong interaction. This is the reason for the fast transfer of electron and fluorescence quenching of the substrate with 2,4-DCP and PA.

**Table 6:** Optimized structures of different organic species (pollutants).

Compound	Optimized structure	Optimized energy (Kcal/mol)
Picric acid		− 112,480.1409
2,4-Dicholorophenol		− 52,852.9565
Benzaldehyde		− 37,734.0778
Aniline		− 30,750.18804

**Table 7:** The interaction of species with ST550 nanostructure. (**a**) ST550 + Picric acid, (**b**) ST550 + 2,4- dichlorophenol, (**c**) ST550 + benzaldehyde, (**d**) ST550 + aniline.

S. no.	Initial geometry	Final optimized geometry	Binding energy (Kcal/mol)
(a)			− 66.21
(b)			− 12.31
(c)			+ 15.98
(d)			+ 35.27

## Conclusions

The ultrasonication method was used in this study to generate mesoporous TiO_2_:SeO_2_ nanostructures which shoes dual application as photocatalyst and fluorophore. XRD, SEM, EDX, TEM, FTIR, UV, and fluorescence spectroscopy were used to characterize the prepared nanostructures. XRD data confirms the presence of anatase phase up to 750 °C, and FE-SEM data shows the formation of nanoparticles with sizes ranging from 28 to 210 nm. The band gap of TiO_2_:SeO_2_ nanostructures was discovered to be 2.16–2.45 eV, which falls within the visible region. The photocatalytic activity of ST550 was optimized and the maximum photocatalytic degradation observed was 90.34% and follows first order chemical kinetics. The nanostructured ST550 was investigated as a fluorescent probe and found to be highly efficient and selective for PA, with a working static quenching mechanism. Stern–Volmer constant, and binding constant, were determined to be 6.31 × 10^–4^ M^−1^, and 5.1844 M^−1^ respectively. The successful application of prepared nanostructure ST550 as fluorophore and light induced photocatalysis for wastewater treatment and purification, future research will be extended to a treatment of organic pollutants diverse groups. The performance of the nanostructure is compared with other photocatalysts reported in literature.

## Supplementary Information

Supplementary Information.

## Data Availability

The datasets used and/or analysed during the current study available from the main authors on reasonable request.
