# Supplementary material for: Exploring the twin potential of nanostructured TiO2:SeO2 as a promising visible light photocatalyst and selective fluorosensing platform
Source: Sci Rep. 2024 Jun 13;14:13677. doi: 10.1038/s41598-024-64167-5 (PMC11637090; doi:10.1038/s41598-024-64167-5)
Supplement: Supplementary file 1 — Supplementary Information. [file 41598_2024_64167_MOESM1_ESM.docx]

**
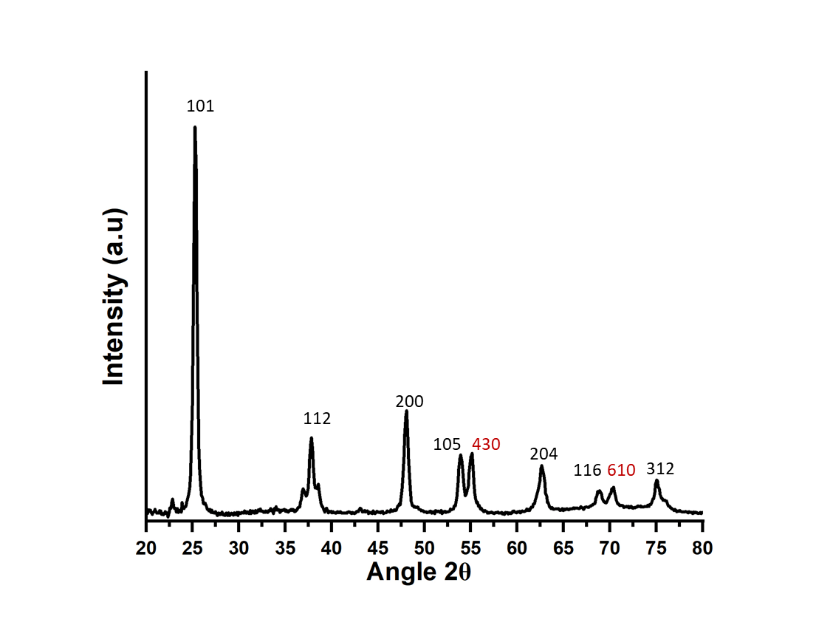
(Supplementary data)**

Figure S1: PXRD pattern of ST2

**Table 1: Crystallite size and lattice strain of photocatalysts calcinated at different temperatures (ST450, ST550, ST750, ST1050, and ST2).**

| Photocatalysts | Crystallite size (nm) | Lattice strain | d- spacing (Å) |
| --- | --- | --- | --- |
| ST450  ST550  ST750 | 90.64  176.62  208.66 | 0.1052  0.0526  0.0449 | 3.5173  3.5185  3.5985 |

**
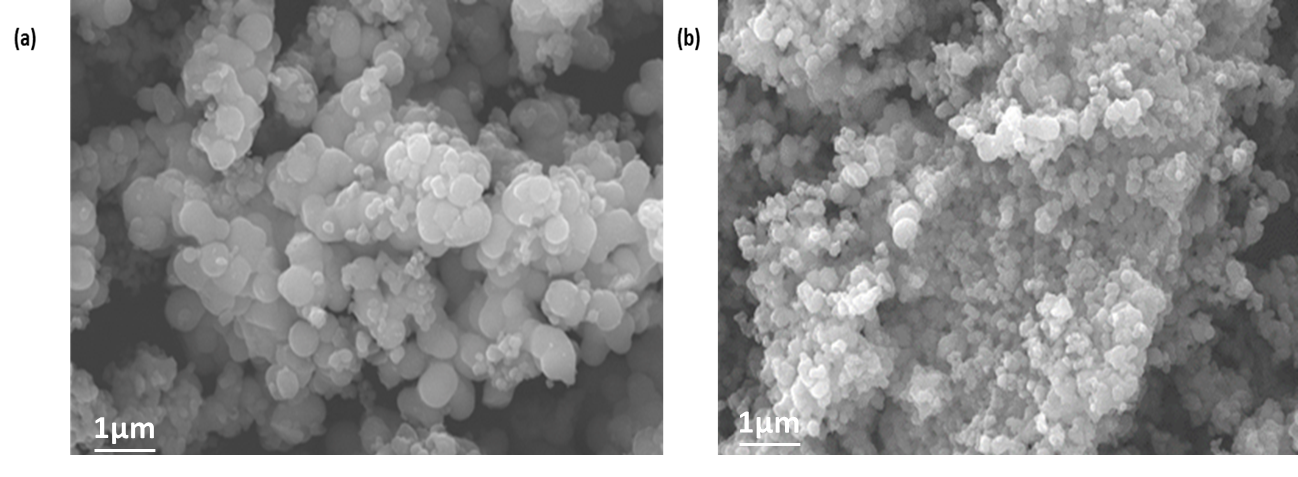
**

**Figure S2 (a-b): SEM image of ST350 and ST750**


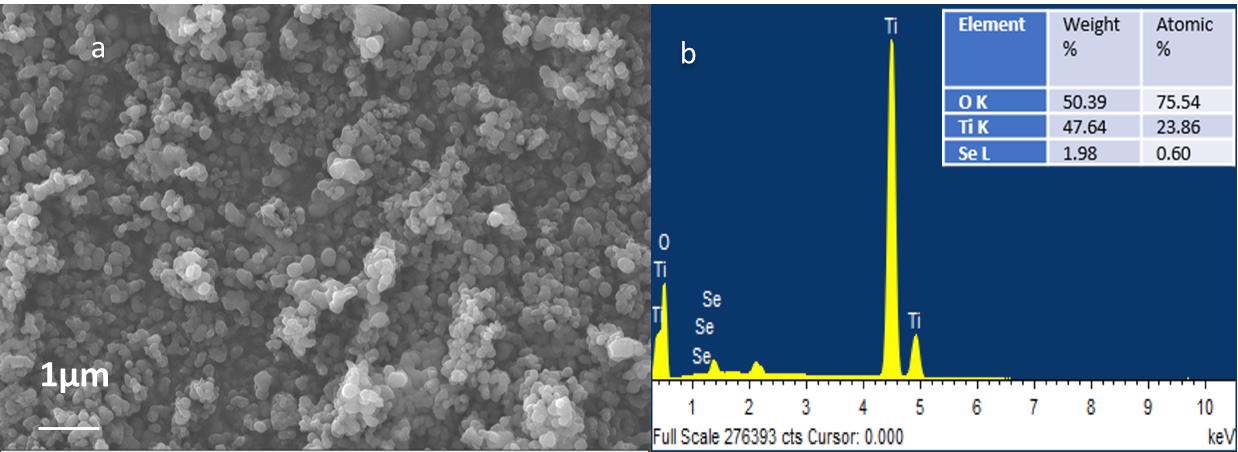


**Figure S3 (a-b): SEM-EDS images of ST2**


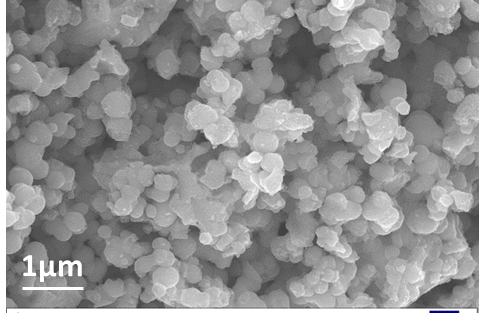


**Figure S4 : SEM image of STB**


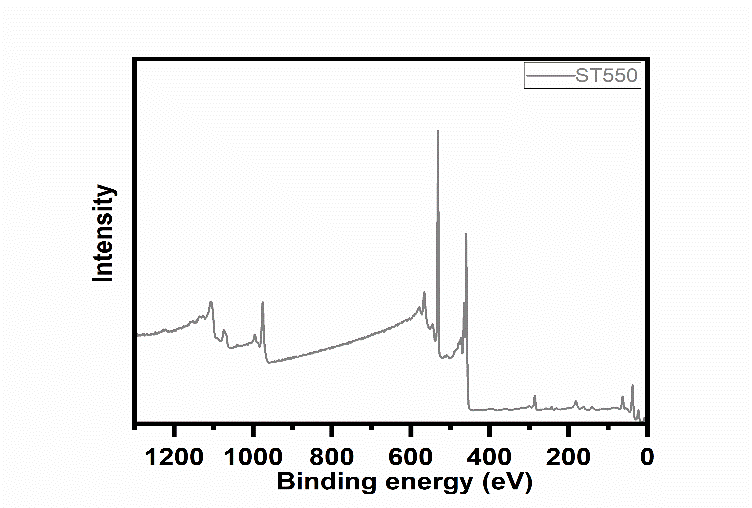


**Se 3d**

**C 1s**

**Ti 2p**

**O 1s**

**Figure S5: Wide scan XPS spectra of ST550**


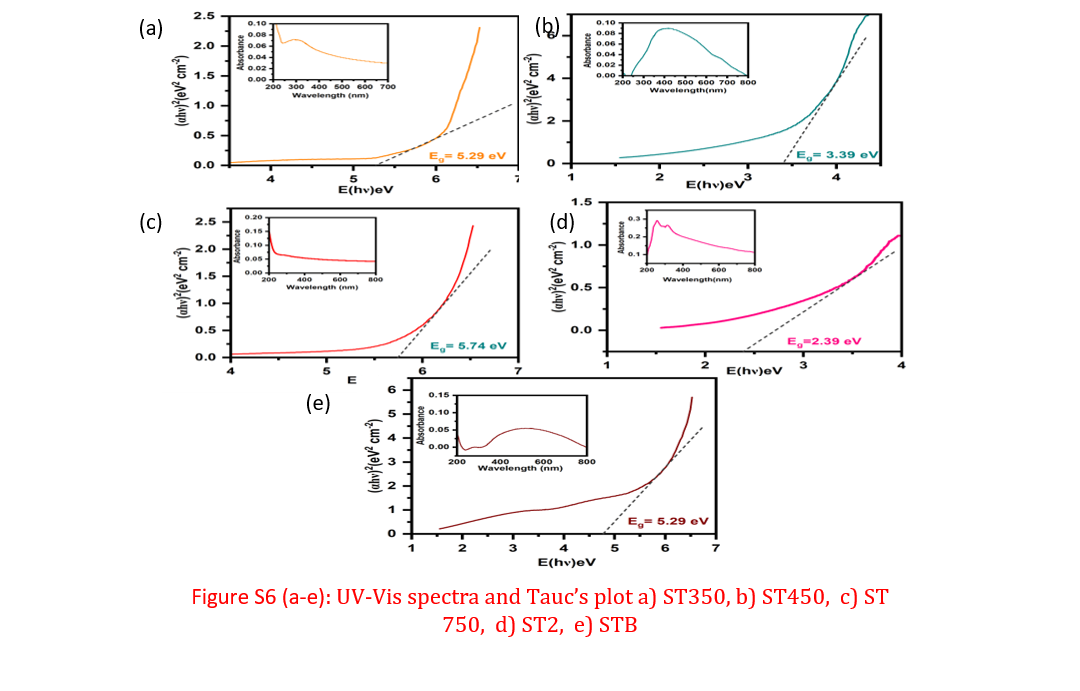


**Figure S6(a-e) : UV-Vis spectra and Tauc’s plot (a) ST350 (b)
 ST450 (c) ST750 (d) ST2 and (e) STB**

b

(b)

(a)


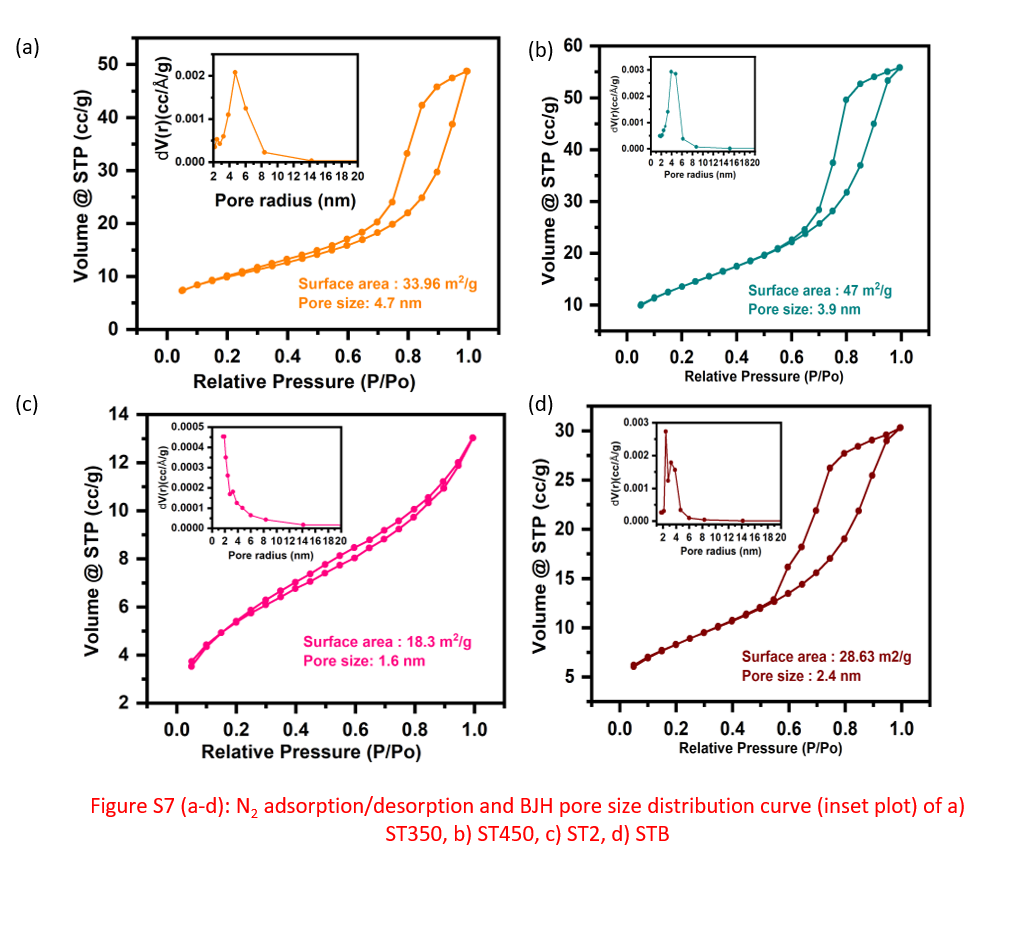


**Figure S7(a-d): N2 adsorption/desorption and BJH pore size distribution curve (inset plot) of (a) ST350 (b) ST450 (c) ST2 (d) STB**
